# Apolipoprotein E Mimetic Promotes Functional and Histological Recovery in Lysolecithin-Induced Spinal Cord Demyelination in Mice

**DOI:** 10.4172/2155-9562.S12-010

**Published:** 2014-04-28

**Authors:** Zhen Gu, Fengqiao Li, Yi Ping Zhang, Lisa B.E. Shields, Xiaoling Hu, Yiyan Zheng, Panpan Yu, Yongjie Zhang, Jun Cai, Michael P. Vitek, Christopher B. Shields

**Affiliations:** 1 Department of Anatomy, Nanjing Medical University, Nanjing, Jiangsu 210029, China; 2 Kentucky Spinal Cord Injury Research Center, Department of Neurological Surgery, University of Louisville School of Medicine, Louisville, KY 40292, USA; 3 Cognosci, Inc. Research Triangle Park, NC 27709, USA; 4 Department of Neurology, Duke University Medical Center, Durham, 27708, NC, USA; 5 Norton Neuroscience Institute, Norton Healthcare, Louisville, KY 40202, USA; 6 Department of Pediatrics, University of Louisville School of Medicine, Louisville, KY 40292, USA

**Keywords:** Focal demyelination, Remyelination, Apolipoprotein E-mimetic, COG112, Oligodendrocyte, Lysolecithin, Inflammation

## Abstract

**Objective:**

Considering demyelination is the pathological hallmark of multiple sclerosis (MS), reducing demyelination and/or promoting remyelination is a practical therapeutic strategy to improve functional recovery for MS. An apolipoprotein E (apoE)-mimetic peptide COG112 has previously demonstrated therapeutic efficacy on functional and histological recovery in a mouse experimental autoimmune encephalomyelitis (EAE) model of human MS. In the current study, we further investigated whether COG112 promotes remyelination and improves functional recovery in lysolecithin induced focal demyelination in the white matter of spinal cord in mice.

**Methods:**

A focal demyelination model was created by stereotaxically injecting lysolecithin into the bilateral ventrolateral funiculus (VLF) of T8 and T9 mouse spinal cords. Immediately after lysolecithin injection mice were treated with COG112, prefix peptide control or vehicle control for 21 days. The locomotor function of the mice was measured by the beam walking test and Basso Mouse Scale (BMS) assessment. The nerve transmission of the VLF of mice was assessed in vivo by transcranial magnetic motor evoked potentials (tcMMEPs). The histological changes were also examined by by eriochrome cyanine staining, immunohistochemistry staining and electron microscopy (EM) method.

**Results:**

The area of demyelination in the spinal cord was significantly reduced in the COG112 group. EM examination showed that treatment with COG112 increased the thickness of myelin sheaths and the numbers of surviving axons in the lesion epicenter. Locomotor function was improved in COG112 treated animals when measured by the beam walking test and BMS assessment compared to controls. TcMMEPs also demonstrated the COG112-mediated enhancement of amplitude of evoked responses.

**Conclusion:**

The apoE-mimetic COG112 demonstrates a favorable combination of activities in suppressing inflammatory response, mitigating demyelination and in promoting remyelination and associated functional recovery in animal model of CNS demyelination. These data support that apoE-mimetic strategy may represent a promising therapy for MS and other demyelination disorders.

## Introduction

Multiple sclerosis (MS) is a neurological disease with presumed autoimmune origin. Its major pathological characteristic is white matter demyelination and secondary axonal loss in the brain and spinal cord resulting from immune cells actively attacking myelin sheaths in the central nervous system (CNS) [1]. The current MS drugs on market focus on modulating immune response and suppressing inflammatory cell infiltration and other inflammatory responses in the CNS without proven activity in addressing existing pathology, i.e., demyelination. Although spontaneous remyelination occurs following relief of a MS episode, it is usually inadequate to reverse worsening symptoms leading to clinical relapse. Therefore, repairing the existing histological damage is required for the MS patients to restore their function and remyelination strategy has drawn extensive attention in the field of MS research and drug development. However, considering the pathogenic complexity of MS, an ideal therapy may be one with combined activities in reducing inflammation and demylination and promoting remyelination.

Apolipoprotein E (apoE) is a 299 amino acid protein with three common human isoforms, namely apoE2, apoE3, and apoE4 [2]. Originally recognized for its role in metabolism and transport of lipid and cholesterol in the cardiovascular system, apoE is also the most abundant apolipoprotein in the nervous system [3] with a major role in supporting and maintaining myelination [4–8]. Following a sciatic nerve crush injury, the synthesis of apoE in the peripheral nervous system (PNS) increased several hundred folds [9], indicating that apoE may be a scavenger of myelin debris following demyelination and may also play a role in delivery of lipids for axonal regeneration and remyelination in the PNS [10,11]. In the CNS, apoE may also maintain homeostasis of cellular lipids [5,12]. Genetic screening in the spinal cord tissue confirmed that apoE is one of the most significantly upregulated genes following spinal cord injury (SCI) [13]. Such intrinsic upregulation of apoE expression may be an auto-reparative mechanism in response to injury.

ApoE is also found to modulate inflammatory responses in the CNS by suppressing microglial activation and inflammatory cytokine release in macrophage/microglia cultures in a dose- and isoform-dependent manner [14–18]. *In vivo* data indicates that apoE plays an isoform-specific role in mediating systemic and brain inflammatory responses [19]. Furthermore, apoE genotype is associated with progression and clinical deterioration of MS [20–22]. Consistently, apoE-knockout mice are more susceptible to and have greater disability in experimental autoimmune encephalomyelitis (EAE), a mouse model of MS [23,24]. Thus, we hypothesize that apoE may represent an ideal target for development of novel therapeutics for MS and other demyelination diseases based on its roles in reducing inflammation and promoting myelination and regeneration. The apoE-mimetic peptide was initially derived from the receptor-binding domain of apoE protein (i.e., apoE133-149) to simulate the bioactivities of the holo-protein [19,25]. COG112 was designed by fusion of apoE133-149 with a protein transduction domain antennapedia (Antp) to enhance blood-brain barrier (BBB) and cell membrane penetration. COG112 has demonstrated more potent anti-inflammatory activity and therapeutic efficacy in EAE mice [24,26]. In the sciatic nerve crush model, systemic administration of COG112 promoted the remyelination and regeneration of peripheral nerves [27]. In the present study, we further elucidate how COG112 affects myelination process in the CNS using an *in vivo* focal demyelination model in mice.

## Materials and Methods

All animal procedures were conducted under protocols approved by the Institutional Animal Care and Use Committee (IACUC) of the University of Louisville. C57BL/6J mice (8–10 weeks old) were obtained from the Jackson laboratory (Bar Harbor, ME) and housed under standard conditions. ApoE-mimetic COG112, and antennapedia (Antp) were synthesized by PolyPeptide Laboratories (San Diego, CA) using standard Fmoc-based chemistry. All peptides were purified by high-performance liquid chromatography (HPLC) to a purity of >95%. The peptide sequence of COG112 is acetyl-RQIKIWFQNRRMKWKKCLRVRLASHLRKLRKRLL-amide. The prefix peptide Antp was found lack of anti-inflammatory activity previously with a sequence of acetyl-RQIKIWFQNRRMKWKK-amide [26].

### Focal Spinal Cord Demyelination Model in mice

**Figure 1:**
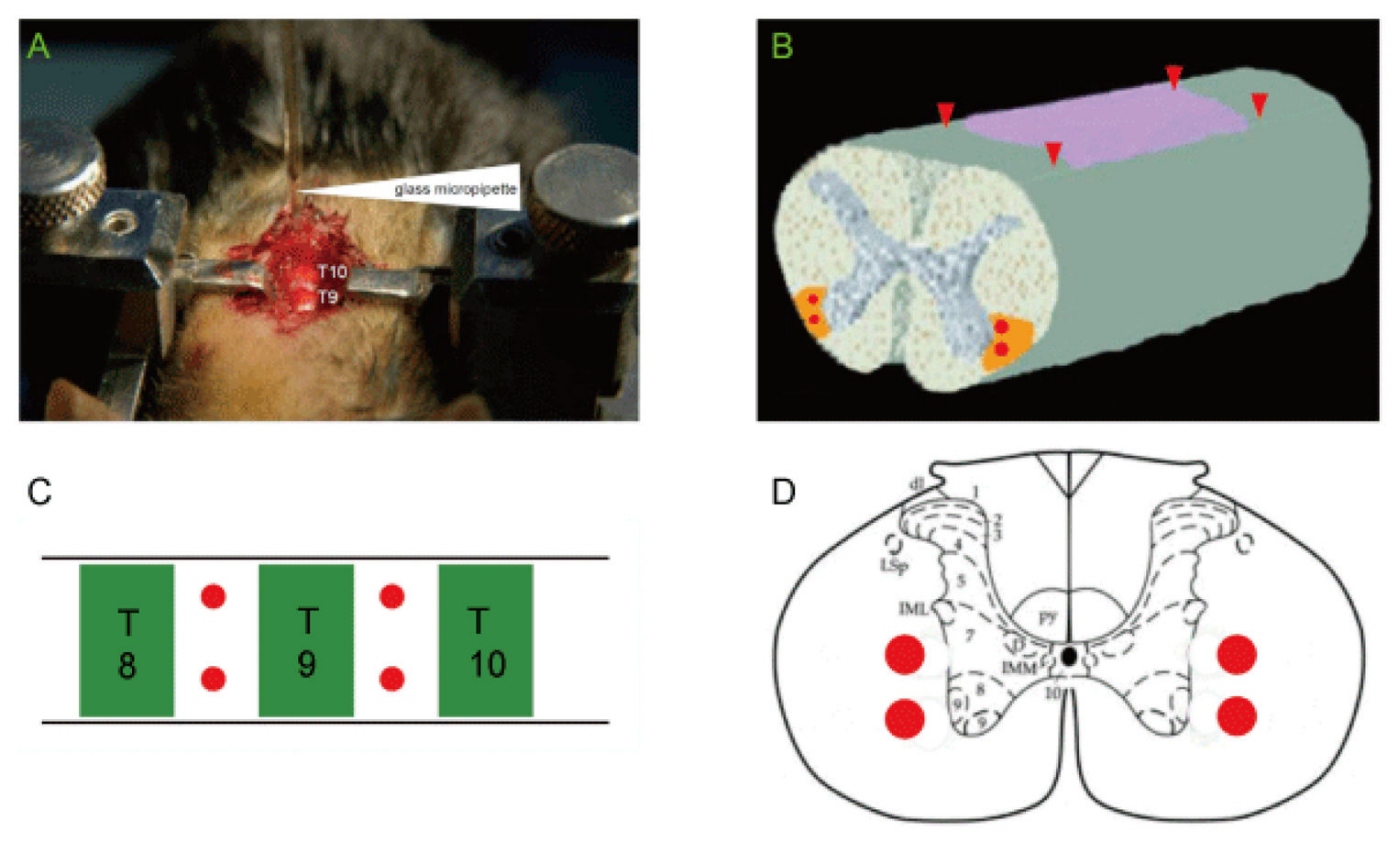
The illustration of spinal cord demyelination lesion in T8 and T9 ventral-lateral funiculus (VLF) on mice. A. The mouse is placed in a U-shaped metal channel and the spinal column was fixed with bilateral stabilizers (top panel). The glass micropipette (indicated by white arrowhead) is positioned over the spinal cord surface (dura removed) under a dissecting microscope. B. Red arrowheads on the steric spinal cord model demonstrate the injection sites and the red dots on the transection plane indicate the lesion sites. C & D. Red dots indicate the injection sites on T8, T9 and T10 from top view (C) and on the transection plane of thoracic spinal cord (D).

Sixty mice were used for this study under anesthesia with a ketamine (100 mg/kg) and xylazine (10 mg/kg) mixture via intraperitoneal injection (i.p.). After immobilizing the thoracic vertebrae with a pair of stainless steel arms of the stabilizer [28], the spaces between T8/9 and T9/10 were exposed by partial laminectomies as illustrated in Figure. 1A. The dura mater overlaying the spinal cord was opened with fine iridectomy scissors to allow a route for stereotaxic injection through a glass micropipette [29]. The tip of the glass pipette was beveled to 30 μm diameter. Lysolecithin [L-α-lysophosphatidylcholine (LPC), Sigma, St. Louis, MO] freshly prepared in phosphate-buffered saline (PBS) (1%) was injected into the ventrolateral funiculus (VLF) of the spinal cord bilaterally at 0.6 mm lateral to the midline and at 0.9 and 1.1 mm depths into the ventral spinal cord with the aid of the Kopf apparatus (Tujunga, CA). In each of the mouse, four spots were stereotaxically injected with LPC at a volume of 0.4 μl per spot by the Nanoject II (Broomall, PA). The injection spots were schematically illustrated in Figure. 1B–D. The sham control animal was injected with an equivalent volume of PBS into the same sites of the spinal cord. The micropipette remained *in situ* for an additional 2 min before withdrawal to prevent solution leakage from backflow. After injection, the mice were released from the stereotaxic apparatus and muscles and skin were sutured in layers. Finally, the animals were moved into the recovery cages containing electric heated blankets (37°C). All mice were given 1 ml saline subcutaneously to prevent dehydration during recovery. The LPC-injected animals were randomly assigned into the following three groups with 15 mice in each: 1) COG112 (2.5 mg/kg/d, i.p.) treatment. 2) Antp-prefix peptide treatment (2.5 mg/kg/d, i.p.), and 3) vehicle control (isovolumic PBS i.p.). COG112, Antp or PBS was injected into the peritoneal cavity immediately after LPC injection.

### Functional Assessment of Mice with VLF Demyelination Lesions of the Spinal Cord

**Figure 2:**
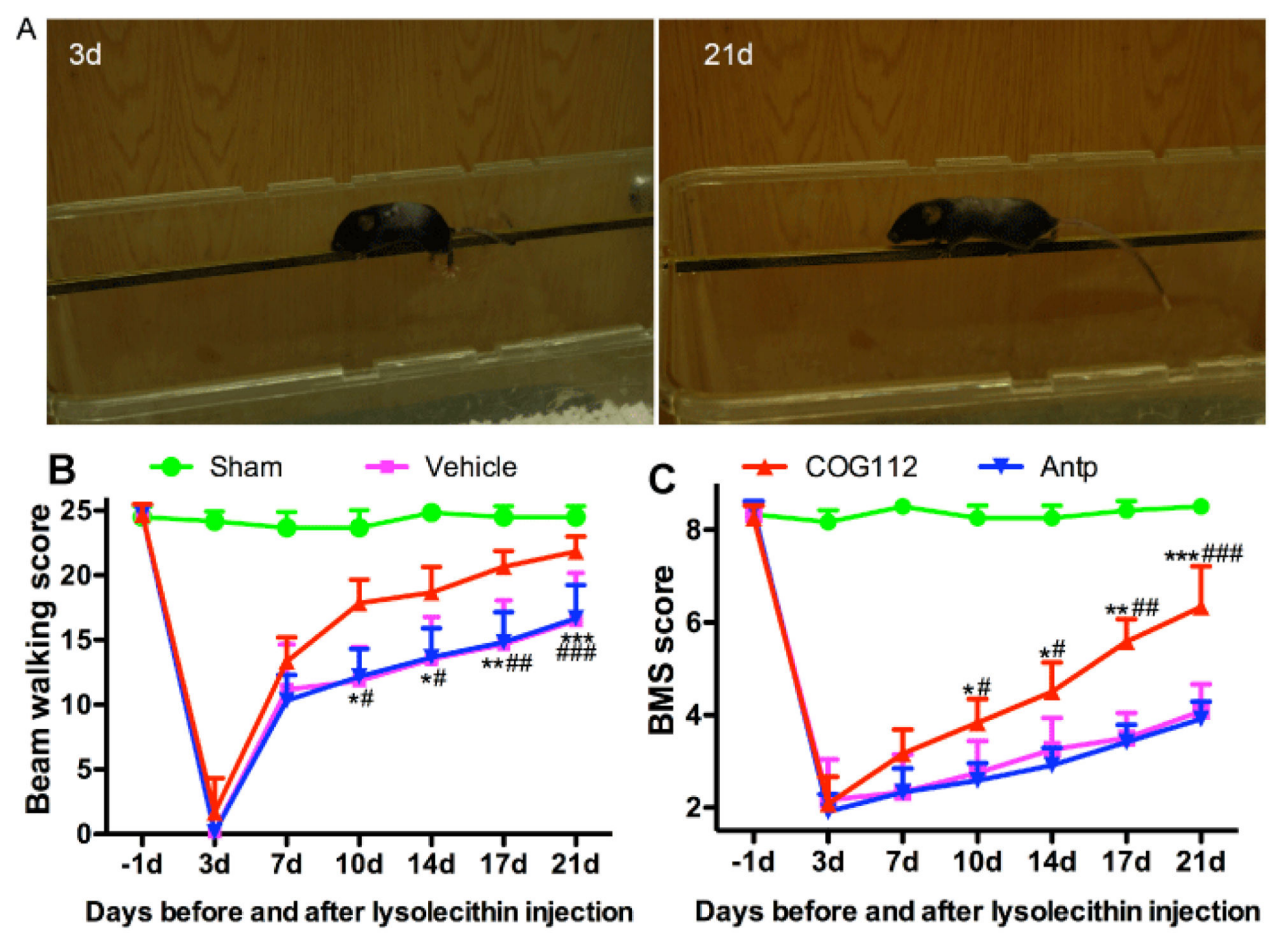
COG112 treatment increases the beam-walking and BMS scores in LPC-induced spinal cord demyelination model in mice. A. Representative pictures to show the great improvement in beam walking following COG112 treatment. B. Beam walking scores, and C. BMS scores were obtained on −1d (before surgery), 3d to 21d (after surgery). The mice were put on the 4 mm beam to walk and recorded with video camera. Note that the forelimbs of the mouse were not affected but the hindlimbs paralysis on the 3rd day while on the 21st day the mouse could hold and stand on the beam with the hindlimbs and keep balance very well. Each value is the mean ± SEM of determinations in 6 mice per group. ^★^, P<0.05; ^★★^, P<0.01; ^★★★^, P<0.001 in COG112 group relative to Antp group. #, P<0.05; ##, P<0.01; ###, P<0.001 relative to vehicle control.

Mice were trained to perform locomotion tests prior to the LPC injection. The Basso Mouse Scale (BMS) and elevated beam walking were tested one day before surgery (−1d) and on the post-surgery days 3, 7, 10, 14, 17, and 21. To reduce subjectivity, two experimenters who were blinded to experimental design were assigned to conduct the behavioral tests independently. For BMS test, mice were placed in an open field and observed for locomotor performance. The BMS score was based on hindlimb movement, body support, stepping, forelimb-hindlimb coordination, and paw or body positioning [30]. For the beam-walking test, a mouse was placed on an elevated metal beam (25 cm long beams with 2, 1.6, 1.2, 0.8, and 0.4 cm widths) as described previously [31] and as demonstrated in Figure. 2. The score was based on the width of the beam and the number of missteps while crossing the beam four times. All animals can cross the 0.4 cm beam without missteps before surgery. After LPC lesion in spinal cord, animals displayed various degrees of deficits in standing as well as crossing the beam. Each hindlimb misstep was recorded as an error. For each mouse, the width of the beam and the number of errors made during beam walking were recorded. The beam walking score was calculated by combining a major score based on beam width and a minor score based on the number of hindpaw missteps, ranging from 0 to 25. A score of 0 indicated the animal’s inability to stand on the beam or dragging its hindquarters on a 2 cm wide beam without body support, and a score of 25 was obtained when the animal was able to walk across a 0.4 cm beam without error. A mouse able to walk on 0.4 cm wide beam typically scored between 21–25, on an 0.8 cm wide beam scored between 16–20, on a 1.2 cm wide beam scored between 11–15, on 1.6 cm wide beam scored from 6–10, and on a 2 cm wide beam scored between 1–5. The numbers of missteps that the mouse made while crossing the beam was subtracted from the highest score associated with the width of the beam crossed. For example, if the mouse misstepped twice while crossing the 1.2 cm wide beam, the score would be 13 (15 − 2 = 13). If the mouse misstepped 3 times while crossing the 0.8 cm beam, the score would be 17 (20 − 3 = 17) [32].

In addition to behavioral tests, animals were also assessed using transcranial magnetic motor-evoked potentials (tcMMEP), an electrophysiological test designed to measure the integrity of the VLF of the spinal cord [28,29,32]. To perform this test, the mouse was restrained in a stockinet and a magnetic stimulator was used to elicit motor evoked potentials, somatosensory evoked potentials, and H-reflexes in non-sedated rodent [31]. The compound muscle action potentials were recorded by placing the active electrode into gastrocnemius, reference electrode into the crural interosseous membrane paralleling to active electrode, and grounding electrode into the base of mouse tail. The transcranial magnetic stimulation (powered by a MES-10 stimulator) was generated through a 5.0 cm coil located on the cranial vertex of the mouse (Cadwell Laboratories; Kennewick, WA). The tcMMEP was induced by a single stimulation at 100% intensity and duplicated for reliability. The latency response in milliseconds (msec) and the peak-to-trough amplitude in millivolts (mV) were recorded which represented the electrophysiological conductivity of the spinal cord.

### Histomorphological assessment

Following functional tests on 21 days after LPC injection into the VLF, the mice were deeply anaesthetized and transcardially perfused with PBS followed by 50 ml 4% paraformaldehyde (PFA). Thoracic spinal cord segments were then dissected and post-fixed in 4% PFA overnight followed by cryoprotection in 30% sucrose. Tissues were frozen in freezing medium (Triangle Biomedical Sciences Inc., Durham, NC), and spinal cords were cut into 20 μm cross sections with a Leica Cryostat (Leica Instruments GmbH, Nusslock, Germany). For immunohistochemistry staining, spinal cord sections were blocked with 10% goat (or donkey serum), 1% bovine serum albumin (BSA), and 0.2% Triton X-100 in PBS for 2 hr at room temperature. Samples were then incubated with primary antibody, 1% goat serum (or donkey serum), 1% BSA, and 0.2% Triton X-100 in PBS overnight at 4?C. The following primary antibodies were used: cluster differentiation 68 (CD68, rat monoclonal 1:500, AbD Serotec Raleigh, NC) and glial fibrillary acidic protein (GFAP, rabbit polyclonal 1:400, Dako, Copenhagen, Denmark). After incubation with primary antibodies, tissues were washed three times in PBS plus 0.05% Triton X-100 at room temperature (at least 1 hour/wash) before being incubated with secondary antibody for 45 min at room temperature. Secondary antibodies include Alexa Fluor 488, Alexa Fluor 546 conjugated goat anti-rabbit, anti-mouse, or anti-rat from Molecular Probes (Eugene, OR); and Texas red, fluorescein isothiocyanate (FITC)-conjugated donkey anti-rat or donkey anti-rabbit from Jackson Lab, Inc. (West Grove, PA). Spinal cord sections were counterstained with 0.1% - 4′, 6-Diamidino-2-phenylindole dihydrochloride (DAPI, Sigma, St. Louis, MO) and were coverslipped with Gel/Mount (Biomeda, Foster City, CA). Imaging was performed using Nikon Ti-U inverted fluorescence microscope coupled by a Nikon NIS-Elements imaging workstation or using Nikon TE2000 microscope equipped with a SPOT imaging system (Diagnostic Instruments, Inc., Sterling Heights, MI). Cell counting and measurement areas were interpreted from the images using software from Image Pro Plus (Media Cybemetics, NY, USA) and Adobe Illustrator (Adobe, San Jose, CA). For eriochrome cyanine (EC) myelin staining, spinal cord sections were dried at 37°C for 30 min and rehydrated in a series of descending ethanol concentrations (100, 95, 80, 70, and 50%), then incubated in 0.2% of FeCl3 and 0.080% of EC in aqueous H2SO4 for 15 min [33]. The sections were washed in distilled water, differentiated in 0.5% ammonia solution for 30 sec and washed again in distilled water, before final dehydration in graded ethanol and coverslipping with Permount (Fisher Scientific, Pittsburgh, PA).

The ultra-structure of the demyelinated spinal cord was examined using an electron microscopy (EM). Three mice of each group were fixed with 4% PFA plus 2.5% glutaraldehyde (Sigma-Aldrich, St. Louis, MO) in 0.1 M PBS (pH 7.4) 21 days after surgery. The thoracic spinal cord at the lesion’s epicenter was removed and similarly fixed overnight at 4°C. Samples were placed in 1% osmium tetroxide (OsO4, E.M.S., Hatfield, PA) and dehydrated in ascending ethanol series and acetone. After embedding in resins (Polysciences Inc., *Warrington*, *PA*), ultrathin sections were cut using an ultramicrotome (LKB, Bromma, Sweden), and each section was collected on individual copper grids. After staining with uranyl acetate and lead citrate, the sections were photographed on a JEM-1010 transmission electron microscope (JEOL, Tokyo, Japan). Sections from each experimental group were subjected to a quantitative evaluation using Image Pro Plus software to determine: (a) the number and density of the axons (per micrometer squared, N/μm2), (b) the axon diameter, axon diameter wrapped with myelin and the g-ratio (diameters of axons/diameters of axons plus myelin). Ten random fields from each experimental group were measured.

### Statistical Analyses

All parametric data were analyzed by one-way ANOVA coupled with Bonferroni post hoc test using GraphPad prism 5.01 (San Diego, CA). Data are expressed as mean ± standard errors of mean (SEM). Difference of BMS score and beam-walking score among groups over time were analyzed by two-way ANOVA followed by Bonferroni post hoc test. A p <0.05 was considered to be significant.

## Results

### ApoE-mimetic promotes functional recovery following spinal cord demyelination

Demyelination of the spinal cord affects hindlimb function, in which the descending and ascending fibers play important roles in initiating and coordinating locomotion [34]. LPC was administrated bilaterally into two segments on each side of the spinal cord to ensure that lesions were created. Three days after the LPC injections, all mice demonstrated dramatic disability in beam-walking performance and significantly reduced BMS scores. Their motor function gradually improved starting from seven days after lesion as depicted in control animals of Figure. 2B & C. The time course of functional recovery was consistent with spontaneous remyelination process as described in previous reports [35]. Compared with slow functional recovery in the vehicle control and Antp groups, COG112 treated animals exhibited significantly expedited functional recovery by both BMS and beam walking assessment (Figure 2). Beam walking scores at 7 days after injury showed that the COG112 group also performed better than the vehicle and Antp groups (Figure 2B). The BMS scores in the COG112 group were significantly higher than those in the Antp group and the vehicle group on days 10, 14, 17, and 21 following the LPC lesion (Figure 2C). These results are consistent with the previous finding that COG112 promotes functional recovery from the disability in EAE models of MS [26].

**Figure 3:**
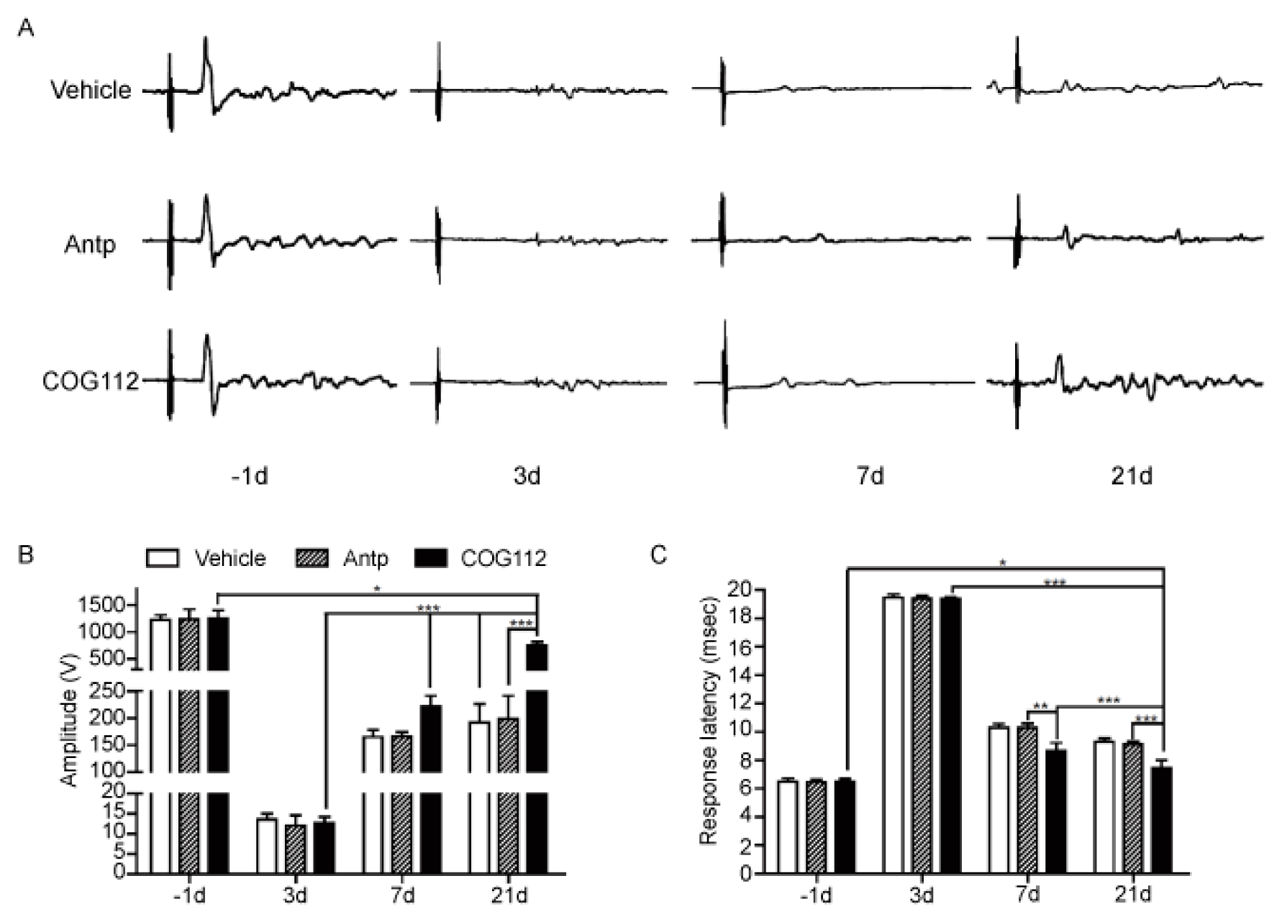
COG112 treatment was associated with increased tcMMEP amplitudes following VLF demyelination in vivo. A) The tcMMEP waveforms of vehicle, Antp and COG112 treated mice on day 1 before surgery (−1d) as well as day 3, 7 and 21 after surgery. B & C) LPC-induced demyelination resulted in a decrease in the tcMMEP amplitude to the background noise level and a delay or no response latency at day 3, with spontaneous recovery in tcMMEP beginning at day 7. The tcMMEP amplitude of COG112 group was significantly greater than those of Antp and vehicle control; while the response latency of COG112 group was significantly shorter than those of Antp and vehicle control. ^★^, P<0.05; ^★★^, P<0.01; ^★★★^, P<0.001 in COG112 group relative to previous time points or vehicle control.

The tcMMEP is a useful electrophysiological assessment of locomotion following spinal cord injury [29,36] as well as maturation of myelinated axons during postnatal motor tract development [37]. In this study, the response of tcMMEPs were delayed or abolished by day 3 following LPC injection and began to recover 1-week later and continued to improve over time as manifested as increased amplitude and shortened response latency of tcMMEP shown in Figure 3. The improvement in electrophysiological response seems to be consistent with the time course of functional gains (Figure 2 and 3). After treatment with COG112 for 3 weeks, the tcMMEPs showed further improvement in amplitudes and shorter response latency compared with those animals exposed to Antp and vehicle controls (Figure 3B & C).

### ApoE-mimetic reduces demyelination area and improves remyelination following LPC injury

**Figure 4:**
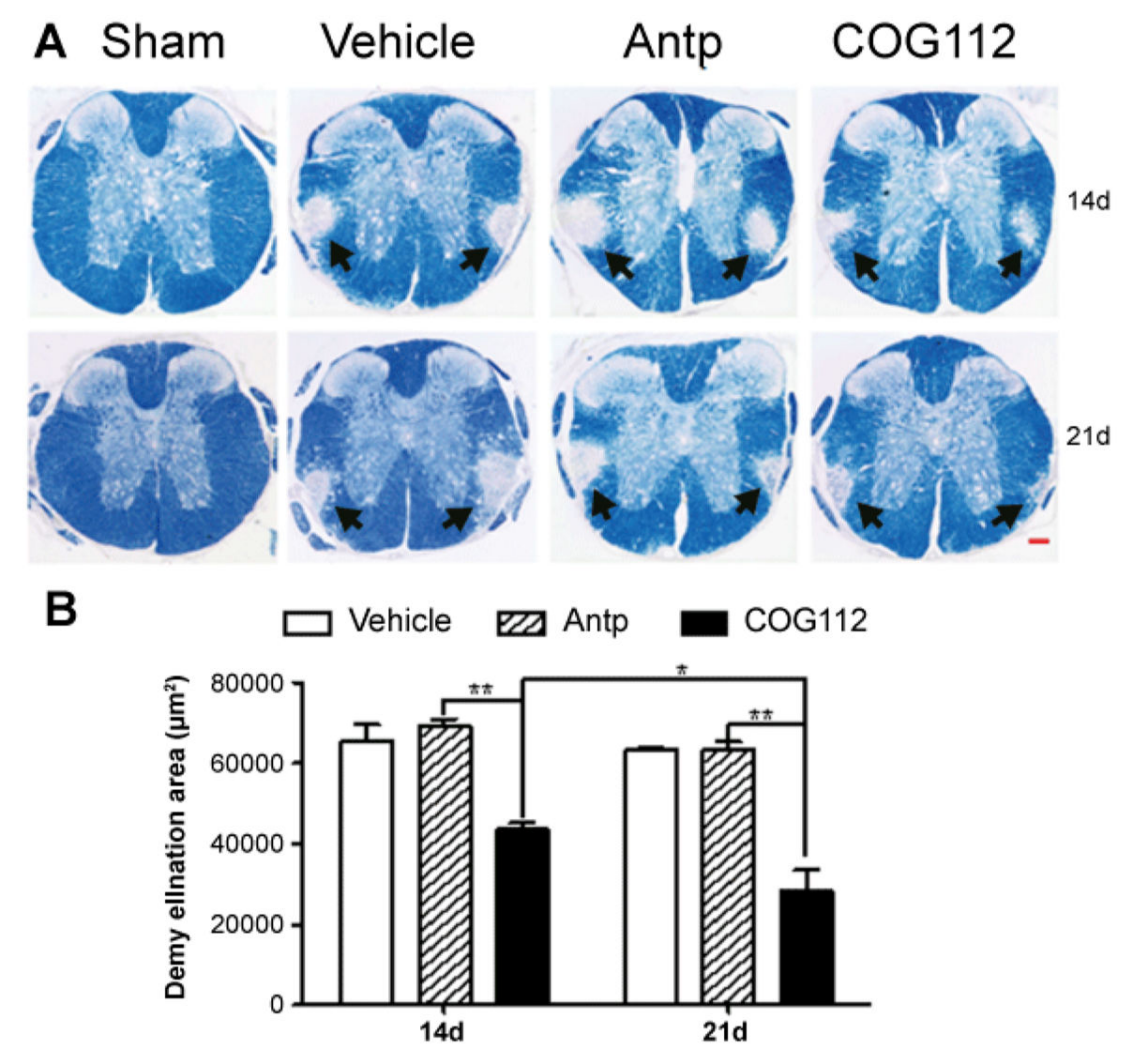
A) Micrographs of spinal cord sections 14 and 21 days after mice were treated with vehicle, Antp and COG112 compared with sham groups stained by eriochrome cyanine (EC). The area of 21 demyelination in the VLF was indicated by arrows (Bar = 50μm). B) The emyelination area was quantified by image analysis. The lesion size in COG112 treated mice was smaller at days 14 and 21 following injury compared with Antp and vehicle groups (mean ± SEM, ^★^ p<0.05, ^★★^ p<0.01, n=6).

To evaluate myelin lesion in the white matter of spinal cord, eriochrome cyanine (EC) staining was used to demonstrate normal white matter stained in dark blue in contrast to the gray matter in light blue. LPC injection into the spinal cord produced well defined areas in the VLF indicating localized demyelination which was not detected in the PBS injection group (i.e., sham group) (Figure 4A). Histopathological analysis showed that the demyelinated lesion size was significantly reduced following treatment with COG112 on days 14 and 21 as compared to the Antp and vehicle groups (Figure 4B).

**Figure 5:**
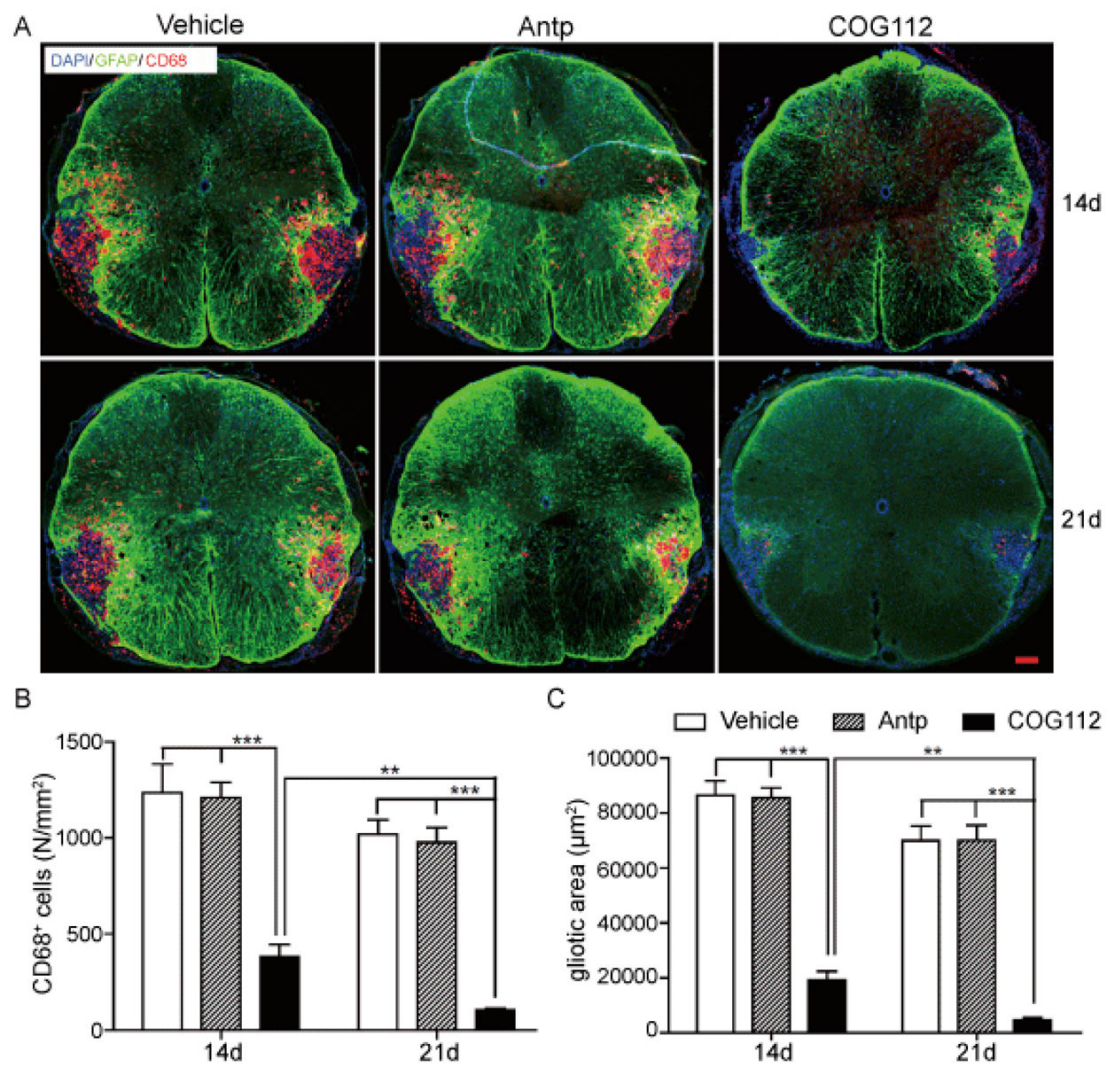
A) Representative CD68 and GFAP immunofluorescence staining of LPC injured spinal cord at 14 d and 21 d mice from vehicle, Antp and COG112 groups (Bar = 100 μm). B) The number of CD68+ cells in lesion area (red) was reduced in the animal treated with COG112. C) The area of reactive gliosis demonstrated by the yellow/green band in the COG112 treated mice were smaller than other groups (mean ± SEM, ^★★^ p<0.01, ^★★★^ p<0.001, n=6).

In addition to the EC staining, a secondary method was used to evaluate demyelination lesion in spinal cord following LPC injection. Astrogliosis was activated by inflammation at the LPC-demyelinated sites where GFAP expression was upregulated. CD68+ activated microglia or macrophages are not detectable in the normal spinal cord, while LPC induced myelin damage increases inflammation and activity of CD68+ cells. CD68+ cells and GFAP+ processes of activated astrocytes accumulating near the area of demyelination to form a discernible band on the immunofluorescence photomicrographs [38]. At day 14 and 21 following LPC injections into the VLF, CD68+ cells were accumulated in the lesion sites and dispersed into adjacent white matter. When compared with spinal cords exposed to vehicle and Antp, the number of CD68+ cells in the COG112 group was significantly lower and GFAP+ bands were smaller at 14 and 21 days (Figure. 5). These results confirmed a role of COG112 in reducing LPC-induced reactive astrogliosis and microglial activation at the lesion site.

**Figure 6:**
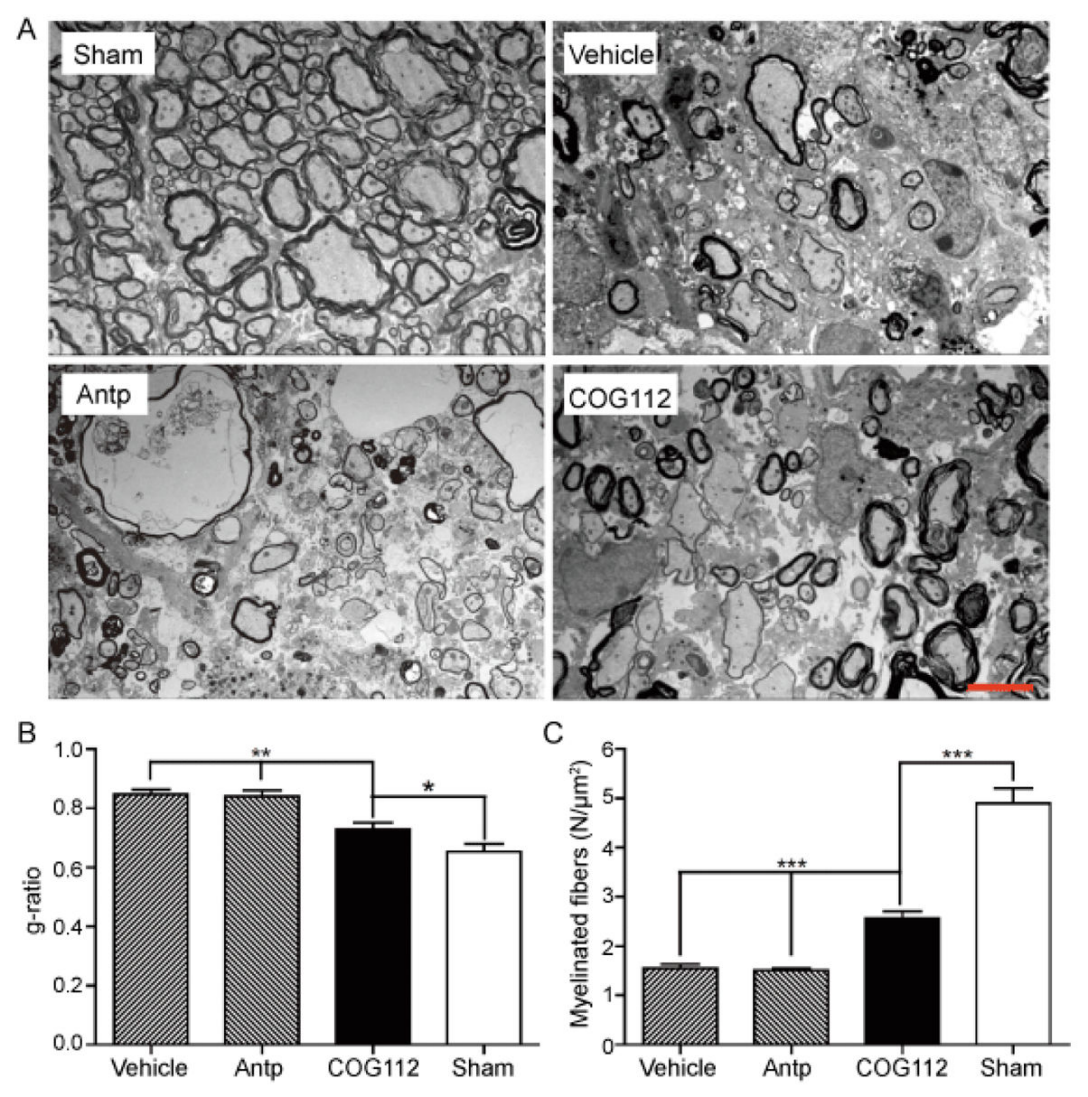
A) Ultrastructure of the spinal cord that compares myelination in mice with and without LPC lesion (sham), and compared to mice with LPC injuries expose to vehicle, Antp and COG112 treatments (Bar = 5 μm). At day 21, the number of thinly myelinated axons are greater in COG112 treated mice than Antp and vehicle groups. B) Measuring the myelinated axons with myelin thickness the mean g-ratios of the axons in COG112 group was significantly smaller than vehicle or Antp treated mice indicative of thicker myelin. C) Injection of LPC reduced axon count in lesion area of vehicle and Antp groups while more fibers were preserved in COG112 group, indicating its role in axon preservation following the injury. (mean ± SEM, ^★^ p<0.05, ^★★^ p<0.01, ^★★★^, p<0.001, n=3).

However, neither the EC staining nor GFAP immunohistochemistry staining could give detailed information in whether the reduced lesion area in COG112 is derived from preventing demyelination or promoting remeylination. Therefore, the ultrastructure of the lesioned area was further evaluated by EM. The VLF region of the spinal cord treated with LPC exhibited robust demyelination. In the lesion epicenter, there were thinly remyelinated axons and unmyelinated axons 21 days after LPC lesion compared with normal VLF (Figure 6A). The g-ratio is the ratio of the diameter of the axon to the diameter of the axon plus the surrounding myelin. On transverse sections of the spinal cord, the myelin sheaths of remyelinated axons were always thinner with a higher g-ratio. The lower the g-ratio, the more healthy the remyelination [39]. Conversely, higher g-ratio of the axon and myelin units indicated thinner myelin sheaths and incomplete remyelination. Compared with the Antp and vehicle groups, g-ratios were significantly lower in animals treated with COG112 (Figure 6B), indicating that the myelin thickness had increased. The health of axons depended on support from their myelin sheaths and associated OLs and, therefore, demyelination may cause axon degeneration if not reversed within a suitable interval. Axon counting in the VLF lesion area showed that the number of axons was higher in COG112 group than in the Antp and vehicle treatment groups (Figure 6C). This finding supports the hypothesis that apoE-mimetic peptide COG112 not only enhanced remyelination but also increased the number of surviving axons in the lesion area.

## Discussion

MS is an inflammatory demyelinating disease of the CNS that results in progressive functional deficits [40,41]. To clarify the pathological mechanisms and develop effective therapies, several animal models have been established. The EAE demyelination model is induced by immunization with myelin oligodendrocyte glycoprotein (MOG) to activate inflammation and demyelination in the white matter of mice [42]. The resulting lesions in the EAE model are multifocal, erratic, and difficult to correlate with functional deficits. In comparison, focal white matter demyelination animal models created by glial toxins offer consistent lesions that are recognized in some forms of MS [43,44]. Hall et al. described demyelination changes induced by exposure to these toxins that present as areas of acute demyelination followed by remyelination involving oligodendrocytes and small numbers of Schwann cells [35]. Well-known demyelinating agents include LPC, diphtheria toxin, ethidium bromide, 6-aminonicotinamide, calcium ionophores, and a combination of anti-galactocerebroside antibody and complement. The demyelinating effect of LPC is reproducible when the agent directly dissolves phospholipids and myelin membranes [45]. Some OLs and astrocytes in the lesion survive which makes the model a more realistic one consistent with demyelination followed by spontaneous repair [43]. The lesion can be quantified because the local LPC treatment produces small, easily distinguishable lesions with well delineated borders, thus allowing for more accurate and reliable measurement [46].

In the present study, in vivo demyelination model induced by LPC was used to test the effect of COG112 on myelin protection, remyelination and functional recovery. We preferred to use the in vivo model of VLF demyelination since it contains long descending and ascending fibers (reticulospinal, raphespinal, ceruleospinal, vestibulospinal, and spinocerebellar tracts) that, when damaged, create severe motor deficits of the hindlimbs which subsequently undergo gradual spontaneous recovery [29]. However, LPC injection into the dorsal column of the spinal cord [33,40,41,47,48], corpus callosum [49], or optic chiasm [50] did not produce measurable functional deficits by conventional methods. In order to ensure VLF demyelination, we injected LPC into two thoracic spinal segments bilaterally and on each side we injected at 2 spots (0.9 and 1.1 mm depths). The 2 spot lesions expanded and converged together to form one big lesion which blocked VLF nerve transmission completely so that the hindlimbs of all mice injected with LPC became paraplegic. The advantage of this model is that functional changes can be objectively measureable by behavior and by tcMMEP electrophysiological method [43]. According to our observation, both the amplitude and the latency of tcMMEP were robustly reduced or abolished following LPC lesion, which might be associated with demyelination. The focal areas of demyelination blocked conduction through the VLF, with the recovery of conduction following remyelination. TcMMEP responses were closely correlated with hindlimb locomotion [51] as well as motor tract myelination [37]. The increased amplitude or shortened response latency of the tcMMEP responses in the COG112 group indicated that more myelinated fibers were available to permit more motor units to discharge and the histological examination by EM as shown in Figure 6 seems support this idea. However, because COG112 were administrated immediately after LPC injection, the improved function in COG112 group may attribute to either its neuroprotective effect against LPC-induced demyelination, or its neurorestorative effect on remyelination or a combination of both.

Together with our previous data, we incline to the view that apoE mimetic support both neuroprotection and neurorestoration. COG112 has demonstrated protective activities previously in the EAE model such as inhibiting the inflammatory cascade, reducing cytokines and free radicals production, and reducing T cell proliferation [26]. In neuroinflammation animal models including cortical demyelination [7], Alzheimer’s disease [30], and peripheral nerve injury [27], COG112 was shown to reduce CD68+ macrophage recruitment [52]. Clarner et al. showed that inflammatory response positively correlate with the magnitude of myelin loss in cuprizone-induced demyelination model [53]. In the LPC-induced demyelination model, we observed that COG112 treatment reduced the inflammatory response and minimized astrocytosis that may hamper myelin repair [35], suggesting neuroprotective property of COG112 plays certain role in this demyelination model. Beyond neuroprotection, COG112 also demonstrated an ability to promote remyelination, rather than solely preserve myelin during the demyelination process. EM analysis shows that some thinly myelinated axons are presented in LPC-lesioned area with a relatively higher g-ratio than sham, indicating these thinly myelinated axons may be newly remyelinated rather than preserved from demyelination. In support of this idea, COG112 was not able to prevent LPC-induced myelin loss in the in vitro cerebral slice culture (unpublished data). Therefore, neuroprotection mechanism of COG112 may render less contribution to the functional and histological recovery in this in vivo model. Taken together, in our study the behavioral and electrophysiological recovery in the LPC-induced demyelination model in mouse spinal cord provide another hint that apoE-mimetic COG112 does promote remyelination in the CNS.

Based on the bioactivities of apoE protein, apoE-mimetic peptide may contribute to remyelination in several ways. First, apoE-mimetic can protect oligodendrocyte precursor cell (OPC) from inflammatory cell death. We have obtained data showing that COG112 can potently suppress microglia activation-mediated inflammatory OPC death in primary mixed glial cultures (Li, et al., unpublished data). Second, myelin debris is accumulated in the demyelination area following MS and SCI and has been recognized as major inhibitor for remyelination and axon regeneration. Therefore, efficient removal of myelin debris will facilitate the process of remyelination. In addition, the rapid utilization of degraded myelin-components into new myelin by OLs may further reduce the debris that contributes to inhibition of OPC differentiation and axonal regeneration [54]. Third, a sufficient supply of cholesterol may be a rate-limiting factor for successful myelination and/or remyelination considering lipids are the main components of myelin sheaths and OLs [55]. Low density lipoprotein (LDL) and very low density lipoprotein (VLDL) receptors are expressed on postnatal OLs in the CNS and are needed for OL maturation [56]. ApoE, a cholesterol transporter, naturally complexes with LDL and VLDL [57] and is taken up by LDL receptors which are selectively expressed on myelinating OLs [56]. In the CNS, apoE is produced mainly by astrocytes [58] and may have general anti-inflammatory effects [59]. Lack of apoE expression in mice was associated with increased inflammation, including induction of several cytokines and proinflammatory responses [59]. ApoE-mimetic peptides are designed according to the LDL receptor-binding domain of apoE and have demonstrated receptor-binding affinity to LDL receptors [60]. Thus, COG112, conjugated with a 17-aminoacid protein transduction domain (PTD)-fragment of antennapedia [26] which enhances the permeation of the blood-brain barrier, may contribute to the myelinating process by delivering LDL and cholesterol more efficiently to the mature OLs. Growing evidence suggests that the ultimate failure of remyelination may not be due to a lack of OPC recruitment but to inhibition of differentiation [54,61]. Obviously, further studies are required to clarify 1) whether apoE-mimetic modulates migration and differentiation of OPCs; 2) how apoE-mimetic affects the clearance and reutilization of myelin debris; and 3) how apoE-mimetic affect cholesterol transportation between different neural cells.

Although the current MS drugs on market suppress demyelination by inhibiting inflammation, there is no evidence indicating that they can repair existing areas of demyelination and axon degeneration. Some treatments focus on the process of early remyelination, such as transplantation of exogenous myelinating cells or precursors that enhances spontaneous repair mechanisms [51]. Myelin is a lipid rich (70 – 85%) structure that influences the clinical status of MS. Various polyunsaturated fatty acids have been tested in animals [62], and ω-6 and ω-3 fatty acid dietary supplementation have been administered clinically in an attempt to enhance remyelination. Whether polyunsaturated fatty acids affect remyelination is unclear, however, lipid availability clearly contributes to myelin repair. Considering the roles of apoE in facilitating lipid uptake by OLs and the reduction of inflammation, apoE-mimetic strategy may represent a novel therapeutic approach for the treatment of demyelinating disorders such as MS and SCI.

## Abbreviations

Antp: Atennapedia, a prefix peptide with a sequence of acetyl-RQIKIWFQNRRMKWKK-amide
apoE: Apolipoprotein E
BBB: Blood-brain barrier
BMS: Basso Mouse Scale
BSA: Bovine serum albumin
CD68: Cluster differentiation 68
CNS: Central nervous system
COG112: Acetyl-RQIKIWFQNRRMKWKKCLRVRLASHLRKLRKRLL-amide
DAPI: 4′, 6-Diamidino-2-phenylindole dihydrochloride
EAE: Experimental autoimmune encephalomyelitis
EC: Eriochrome cyanine
EM: Electron microscopy
FITC: Fluorescein isothiocyanate
GFAP: Glial fibrillary acidic protein
HPLC: High-performance liquid chromatography
IACUC: Institutional Animal Care and Use Committee
i.p: intraperitoneal injection
LDL: Low-density lipoprotein
LPC: Lysophosphatidylcholine
MOG: Myelin oligodendrocyte glycoprotein
MS: Multiple sclerosis
OLs: oligodendrocytes
OPC: Oligodendrocyte precursor cell
PBS: Phosphate-buffered saline
PFA: Paraformaldehyde
PNS: Peripheral nervous system
PTD: Protein transduction domain
SCI: Spinal cord injury
tcMMEP: Transcranial magnetic motor-evoked potentials
VLDL: Very low density lipoprotein
VLF: Ventrolateral funiculus
